# Synthesis and Characterization of a Novel Composite Scaffold Based on Hyaluronic Acid and Equine Type I Collagen

**DOI:** 10.3390/pharmaceutics14091752

**Published:** 2022-08-23

**Authors:** Erwin Pavel Lamparelli, Veronica Casagranda, Daniele Pressato, Nicola Maffulli, Giovanna Della Porta, Davide Bellini

**Affiliations:** 1 Laboratory of Translational Medicine, Department of Medicine, Surgery and Dentistry, University of Salerno, Via S. Allende, 84081 Baronissi, Italy; 2 Novagenit Srl, Viale Trento 115/117, 38017 Mezzolombardo, Italy; 3 Centre for Sport and Exercise Medicine, Barts and The London School of Medicine, Queen Mary University of London, London E1 4NL, UK; 4 Research Centre for Biomaterials BIONAM, Università di Salerno, Via Giovanni Paolo II, 84084 Fisciano, Italy

**Keywords:** biomaterial synthesis, 3D scaffold, cartilage implant, hyaluronic acid, collagen, reaction in heterogeneous phase, cytotoxicity studies, mechanical behavior

## Abstract

Herein, the synthesis and characterization of a novel composite biopolymer scaffold—based on equine type I collagen and hyaluronic acid—were described by using a reaction in heterogeneous phase. The resulting biomimetic structure was characterized in terms of chemical, physical, and cytotoxicity properties using human-derived lymphocytes and chondrocytes. Firstly, FT-IR data proved a successful reticulation of hyaluronic acid within collagen structure with the appearance of a new peak at a wavenumber of 1735 cm^−1^ associated with ester carbonyl stretch. TGA and DSC characterizations confirmed different thermal stability of cross-linked scaffolds while morphological analysis by scanning electron microscopy (SEM) suggested the presence of a highly porous structure with open and interconnected void areas suitable for hosting cells. The enzymatic degradation profile confirmed scaffold higher endurance with collagenase as compared with collagen alone. However, it was particularly interesting that the mechanical behavior of the composite scaffold showed an excellent shape memory, especially when it was hydrated, with an improved Young’s modulus of 9.96 ± 0.53 kPa (*p* ≤ 0.001) as well as a maximum load at 97.36 ± 3.58 kPa compared to the simple collagen scaffold that had a modulus of 1.57 ± 0.08 kPa and a maximum load of 36.91 ± 0.24 kPa. Finally, in vitro cytotoxicity confirmed good product safety with human lymphocytes (viability of 81.92 ± 1.9 and 76.37 ± 1.2 after 24 and 48 h, respectively), whereas excellent gene expression profiles of chondrocytes with a significant upregulation of SOX9 and ACAN after 10 days of culture indicated our scaffold’s ability of preserving chondrogenic phenotype. The described material could be considered a potential tool to be implanted in patients with cartilage defects.

## 1. Introduction

Connective tissue lesions (e.g., articular cartilage) represent a serious problem for public health and their treatment with advanced biomaterials is becoming a prominent therapeutic option in clinical settings [1]. For regenerative medicine, the key aims are to enhance the activity and viability of cells in injured tissues to encourage natural healing. As it is well-known, cells are generally cultured and expanded in vitro using artificial conditions (e.g., polystyrene dish or flask, monolayer, etc.) that are very different from physiological in vivo conditions which can therefore lead to low cell activity, owing to of reduced cell–cell and cell–ECM interactions. The use of suitable biomaterials, analogous to ECM, can be a way to overcome this issue. Indeed, biomaterials, especially when composed of natural components of ECM, can help to enhance cell activity or functions and regulate immune response advantageously [2,3]. However, one of the most critical aspects is the lack of biomaterials with suitable mechanical properties, low susceptibility to enzymatic catabolism, and proper chondro-integration process [4]. In this context, the synthesis of novel biomaterials is important to develop new surgical systems for in vivo implants [5,6]. Typically, this is achieved using biodegradable and biocompatible materials, which may result from natural or synthetic sources [7]. For instance, alginate hydrogel scaffolds provide a convenient means to preserve cell phenotypes of connective tissue, including chondrocytes, along in vitro expansion [8]; however, their clinical application may be limited by the potential calcification of the constructs [9].

Conversely, collagen-based scaffolds have been described as more appropriate for supporting chondrocytes’ or stem cells’ adhesion and proliferation, being more like the natural extracellular matrix (ECM) compared with other materials [10]. These collagen-based scaffolds would also comply with all the relevant safety standards [11]. Furthermore, this biomaterial is already applied in the clinical setting as a scaffold not only for growing autologous chondrocytes in the MACI (Matrix-induced Autologous Chondrocyte Implantation) procedure, but also for the regeneration of other connective tissues.

On the other hand, collagen is the most common structural protein of connective tissues in mammals and is involved in several biological functions, including support and nourishment [12]. Currently, collagen scaffold mainly comes from animal sources and exists in different physical forms—such as sponges, films, and hydrogels [13]. However, this last one seems to be the most effective in ensuring homogeneous cell adhesion and proliferation [14]. The only drawback is represented by low mechanical strength that is unsuitable for a possible in vivo implantation [15]. Additionally, gelatin—a product derived from thermal denaturation or hydrolysis processes of collagen [16]—presents the same poor mechanical properties and is also more susceptible to proteases compared to collagen [17]. These properties limit its clinical application and they are responsible for its faster in vivo degradation rate. 

Another important consideration is that, despite there being many isoforms of collagen, only a few are used to produce biomaterials aimed at orthopaedic surgery. Type I collagen is currently the gold standard in the field of regenerative medicine [18], indeed, it is preferred with respect to type II collagen owing to its simpler and quicker isolation, greater availability, as well as a lower in vivo immunogenicity [19]. The common sources of collagen include equine tendons, bovine skin, and rat tails. A way to increase mechanical and enzymatic resistance of collagen-based scaffolds is to use a chemical crosslinking reaction. Glutaraldehyde and its derivatives are most often employed for this purpose [20]. However, these reagents showed considerable toxicity on cells, which has discouraged their use [21]. 

An alternative strategy to improve collagen scaffolds’ mechanical and enzymatical properties consists in the development of hybrid scaffolds, obtained by the combination of different biomaterials. Recently, attention has focused on the possibility to incorporate different glycosaminoglycans (GAGs) in collagen matrices, thus obtaining biomimetic composite scaffolds with good porosity [22,23]. Among GAGs, HA seems to be the most promising. From a chemical point of view, HA is an anionic linear non-sulfated polysaccharide of natural origin, commonly found both in the ECM of connective tissues and synovial fluid of joints, where it helps to reduce friction between bone cartilages [24]. A combination of GAGs and collagen was found more helpful and effective for cartilage regeneration [25]; indeed, Mohammadi et al. have reported the preparation of hybrid scaffolds containing type I collagen derived from rat tail and hyaluronic acid (HA) at different ratios, confirming the better physical and chemical performance of blend scaffolds compared with single components [22]. Tang and Spector also investigated the incorporation of different amounts of HA into type I collagen derived from bovine tendon and their findings demonstrated that HA can play a key role in enhancing the collagen mechanical behavior and in vitro chondrogenesis [26]. Previous studies have further highlighted that HA is able to induce chondrogenesis of human adipose-derived mesenchymal stem cells (hAD-MSCs) thanks to the interaction with CD44, a glycoprotein expressed on the cell surface acting as a receptor [27]. 

To date, one of the major problems concerning the synthesis of hybrid scaffolds based on collagen/HA is represented by the mixing of HA within the collagen solution. Although reactions in the homogeneous phase are more advantageous in terms of yield and control, they are very difficult to set up owing to the different stability between HA and collagen; indeed, while collagen shows good solubility and stability in acidic aqueous solutions (e.g., acetic acid), the same acidic environment—especially if it is lower than pH 4—can determine HA degradation, thus leading to a change in original molecular weight. The pH-induced degradation of HA is mainly ascribed to the cleavage of glycosidic bonds among monosaccharides [28]. On the other hand, increasing hyaluronic acid molecular weight and concentrations leads to a rise in solution viscosity, which prevents its homogeneous incorporation [29]. Another drawback is that in aqueous environments collagen and hyaluronic acid form polyion complexes (PIC), being respectively a cationic polypeptide and an anionic polysaccharide, thus forming scaffolds inhomogeneous in structure and with poor mechanical properties [30].

To overcome this, a potential solution from a chemical synthesis point of view is the incorporation process by heterogeneous phase [31]; this approach could improve the integration of a greater amount of HA and guarantee the maintenance of the original porosity of collagen sponges. It should be noted that porosity is also required to ensure a correct exchange of oxygen and nutrients between cells and the surrounding environment. Taking into account these considerations, a novel composite scaffold formed by equine type I collagen and hyaluronic acid was synthesized and characterized. Fourier transform infrared (FT-IR) spectroscopy and thermal analysis were adopted to check the synthesis product; scaffold morphology and its mechanical properties were documented respectively by field emission scanning electron microscopy (FE-SEM) and tensile test. Finally, in vitro enzymatic degradation, viability assay on human peripheral blood mononuclear cells (hPBMCs), and chondrocyte cultures coupled with RT-qPCR assays were performed to evaluate both scaffold toxicity and its potential use as a biomaterial for surgery implants.

## 2. Materials and Methods

Commercial equine type I collagen sponge was purchased from Euroresearch (Milan, Italy). Sodium hyaluronate (molecular weight 0.25–0.45 MDa) was purchased from Contipro Pharma (Dolní Dobrouč, Czech Republic); Collagenase from *Clostridium hystolyticum* was purchased from Sigma-Aldrich (Milan, Italy). All other chemicals were reagent grade from Sigma and Fluka. All chemicals were used without further purification unless otherwise specified.

### 2.1. Synthesis of the Composite Scaffold

To synthesize the composite scaffold, here called ‘HyCo’, a commercial equine type I collagen sponge (Euroresearch—Milan, Italy), in lyophilized form, was previously hydrated with a water solution of tetrabutylammonium salt of hyaluronic acid (HA-TBA). This last reagent was synthesized using a cationic exchange process, starting from the sodium salt of HA, as better described elsewhere [32].

In more detail, a square-shaped plaque with measuring 16 × 16 cm—with a thickness of 5 mm, and a weight of 2.0 g—was placed in contact with 100 mL of an aqueous solution, containing 1% (*w/v*) of HA-TBA, until complete absorption. After that, the wet membrane was first frozen at a temperature of −40 °C for 24 h and subsequently lyophilized using a laboratory freeze dryer (ScanVac CoolSafe 55-9 Pro; LaboGene ApS, Lynge, Denmark) to obtain the dry intermediate product. Therefore, the resulting co-lyophilized membrane has undergone a chemical crosslinking reaction (continuous boiling under reflux for 8 h) in the heterogeneous phase using acetone as solvent, for activating terminal carboxyl groups of the hyaluronic acid (HA), as described detailing in the registered patent by Novagenit Srl (patent application no. 102018000005455). This rection promoted the formation of amide and ester bonds among the carboxyl groups of HA and amine and hydroxyl groups of the type I collagen in order to entrap HA within the collagen matrix for avoiding its dissolution in aqueous media. The crosslinked membrane has undergone a purification phase by washing with different solutions to ensure the removal of solvent residual. The cleansed membrane was finally freeze-dried, packed in medical paper, sterilized by beta irradiation (25 kGy) in a GLP-certified factory (STERIS S.p.A., Seriate BG, Italy), and stored at 4 °C temperature until its use.

A schematic representation of the whole process is shown in Figure S1. The percentage of HA into the final product was around 25% *w/w* and it has been derived gravimetrically, considering the difference in weight between the crosslinked scaffold and starting collagen scaffold.

### 2.2. FTIR Analysis

FTIR analysis was performed with FT-IR Agilent Cary 630 Spectrometer (Agilent Technologies, Santa Clara, California, CA, USA) equipped with an attenuated total reflection (ATR) diamond unit. In detail, roughly 100 mg of the dry sample of each scaffold (HyCo and collagen alone) was tested in wavenumber range from 650 to 4000 cm^−1^ (30 scans with a resolution of 4 cm^−1^, 15 s per sample, and three replicates per sample) with the program MicroLab PC (Agilent Technologies, Santa Clara, CA, USA). The peaks corresponding to specific functional groups were identified in final infrared spectra and then compared with those reported in the literature.

### 2.3. TGA and DSC Analysis 

Thermogravimetric analyses (TGA) were carried out in an air atmosphere with a Mettler Toledo TC-10 thermobalance from 25 °C to 700 °C at a heating rate of 10 °C/min. Differential scanning calorimetry (DSC) analyses were carried out in N_2_ atmosphere with a Mettler Toledo DSC 822e from −50 °C to 160 °C at a heating rate of 10 °C/min.

### 2.4. Morphological Characterization by FE-SEM

Surface and section morphology of scaffolds were observed by field emission-scanning electron microscopy (FE-SEM) (mod. LEO 1525; Carl Zeiss SMT OG, Oberkochen, Germany). Samples were first immersed in liquid nitrogen until freezing, and then fractured with a needle in order to expose their internal surface, placed on a double-sided adhesive carbon tape previously glued to an aluminum stub, and coated with a gold film (250 A thickness) using a sputter coater (mod. 108 A; Agar Scientific, Stansted, UK), before observation.

### 2.5. Tensile Test

Mechanical behaviors of all samples were measured in a tensile way at room temperature according to the guidelines established by the Standard Test Method for Tensile Properties of Polymer Matrix Composite Materials (ASTM D3039/A3039M) using an Instron 5967 machine with Bluehill software (Norwood, MA, USA) equipped with a 10 N load cell. Samples were shaped to obtain specimens having a gauge length (Lo) of 22 mm and a width (W) of 10 mm; sample thicknesses (S) ranged from 3 to 5 mm. A monoaxial deformation was applied to the sample with a speed of 10 mm/min, and force (F) and elongation (L) during traction was registered. The value of force (F) provided by the instrument was divided by the sample area (A = W × S) to obtain the strength values (σ = F/A). The deformation values (L) during the run were compared to the initial length to obtain values of strain (ε = (L − Lo)/Lo); the ultimate tensile strength (σ max, expressed in kPa) was calculated as load to failure/cross sectional area of the sample.

### 2.6. Absorbency and Swelling Ratio Assays

Each scaffold was cut (Swann-Morton^®®^, Sheffield, England) to obtain three samples with dimensions of 5 × 5 cm and a thickness of 3 mm (HyCO) and 4 mm (collagen). Samples were weighted by an analytical balance (Model LE 324S-Sartorius, Gottingen, Germany) and their dry weights registered; moreover, the volume (V) of each sample was calculated, considering their initial length, width, and thickness. Each scaffold was placed in a Petri dish and hydrated with a volume of preheated (37 °C) saline aqueous solution (containing 142 mM of NaCl and 2.5 mM of CaCl_2_) equivalent to 40 times the sample’s mass. At this stage, all samples were transferred in a standard incubator (37 °C, 5% carbon dioxide (CO_2_), and 85% relative humidity (RH)) for mimicking physiological conditions. The difference in mass, before and after the hydration phase, was registered and the resulting absorption capability (mg/mm^3^) was calculated using the Equation below (1). Absorbency was measured at two time intervals: 30 min and 24 h.

(1) $$\mathrm{Absorbency}\;(\mathrm{mg}/{\mathrm{mm}}^{3})=\frac{W\left(\mathrm{wet}\right)\;–\;W\left(\mathrm{dry}\right)}{V}$$

where W(wet) is the weight of the sample after different hydration times while W(dry) is starting raw weight, and finally V is the initial volume of the respective specimen. At every scheduled time point, the matching samples were collected and suspended by one corner with tweezers for 30 s in order to remove the excess solution by gravity, and then finally carefully reweighed [33].

Moreover, swelling behavior of the different scaffolds in phosphate-buffered saline (PBS) solution (pH 7.4) at 37 °C for 24 h was monitored. To achieve this, the lyophilized samples were weighed and then placed in PBS at 37 °C. The swelling ratio (%) was calculated using the following Equation (2)

(2) $$\mathrm{Swelling}\;\mathrm{ratio}\;(\%)=\frac{W\left(\mathrm{wet}\right)\;–\;W\left(\mathrm{dry}\right)}{W\left(\mathrm{dry}\right)}\;\times \;100$$

where W(wet) is the weight of the sample after different hydration times and W(dry) is starting raw weight. All assays were carried out in triplicate and, for each, the mean value and the standard deviation were reported.

### 2.7. In Vitro Enzymatic Degradation Assay

The in vitro degradation of the two scaffolds was performed using a bacterial type I collagenase from *Clostridium hystolyticum*, according to a protocol described elsewhere [34]. The samples (about 40 mg) were accurately weighed (W_0_) following a vacuum drying cycle (24 h) for removing residual moisture, hydrated with 4 mL of 0.1 M Tris–HCl (pH = 7.4), containing 5 mM CaCl_2_ and 50 mg/L NaN_3_ and then placed at 37 °C. After one hour of incubation, 4 mL of collagenase solution (2.4 U/mL) in Tris–HCl (pH 7.4, 37 °C) were added to give a final collagenase concentration of 1.2 U/mL. Degradation was evaluated at scheduled time intervals (24, 48, and 72 h) by stopping the collagenase activity with the addition of 1 mL of 0.25 M Na_2_-EDTA to each sample. A gravimetrical method was used to determine the weight-loss of the samples: following the action of collagenase for 24, 48, and 72 h, residual samples for each time-point were washed three times with distilled water before freeze-drying and weighed again (W_t_). The extent of degradation was expressed as percentage of the original weight of the samples (see Equation (3)) and reported in function of the degradation time.

(3) $$\mathrm{Weight}\;(\%)=\frac{\mathrm{Weight}\;\mathrm{time}-\mathrm{point}}{\mathrm{Weight}\;\mathrm{time}-\mathrm{zero}\;}\;\times \;100$$

where Weight (time–point) is the weight of the sample after the action of collagenase at scheduled time intervals (24, 48, and 72 h) and Weight (time–zero) is starting raw weight following the vacuum drying cycle (24 h) for removing residual moisture. All tests were performed in triplicate and standard deviations were reported.

### 2.8. Cells Harvesting and Cytotoxicity Assay

Human peripheral blood mononuclear cells (hPBMCs) were obtained from Buffy Coats donated from University Hospital Blood Bank, as anonymous material from healthy donors and separated by Ficoll-Hypaque gradient density (Sigma-Aldrich) following standard techniques [35]. After isolation, cells were re-suspended in RPMI medium, supplemented with 10% heat-inactivated FBS, 10 mg/mL L-glutamine, and penicillin/streptomycin (50 U/mL). Cells were then seeded in 12-well plates at a density of 1 × 10^6^ cells/mL and incubated in a humidified atmosphere containing 5% CO_2_ and 95% air. Cell metabolic activity was analyzed using the MTT assay. Briefly, cells were incubated along with membranes (concentration about 6.7 mg/mL) for 24 and 48 h using porous transwells (3 µm) (Greiner bio-one, no. 665631) in order to maintain cell separation (in suspension) from membranes. Adopting this protocol, the cells and membranes are still in contact through the culture medium but the cell migration and their resulting diffusion within scaffold pores are prevented. Following treatment, 3-(4,5-Dimethylthiazol-2-yl)-2,5-diphenyl-tetrazolium bromide (MTT) was added (1 mg/mL) to each well and incubated at 37 °C for an additional 4 h. Plates were centrifuged at 300 g for 10 min and the supernatants were completely removed. Formazan products were dissolved in dimethyl sulfoxide (DMSO). Absorbance was determined at 570 nm using a microplate reader (Infinite F200 PRO, Tecan Group Ltd., SW). All assays were performed in triplicate (N = 3). For the MTT assay on hPBMCs, three independent experiments were performed in triplicate, each one on a single subject (N = 3). Cell metabolic activity, a surrogate of viability, was calculated as the percentage of the control group, considered as 100%. The percentage viability of cells was calculated according to Equation (4):

(4) $$\mathrm{Cell}\;\mathrm{metabolic}\;\mathrm{activity}\;(\%)=\frac{\mathrm{Abs}\;\mathrm{of}\;\mathrm{sample}-\mathrm{Abs}\;\mathrm{of}\;\mathrm{blank}}{\mathrm{Abs}\;\mathrm{of}\;\mathrm{control}-\mathrm{Abs}\;\mathrm{of}\;\mathrm{blank}}\;\times \;100$$

Human chondrocyte line (Cell Applications, Inc., San Diego, CA, USA, Lot 2672) was cultured on HyCo membrane for 28 days and cell viability was determined by an adenosine-5′-triphosphate (ATP) assay with the Promega CellTiter-Glo^®®^ 3D Cell Viability kit (Promega Italia S.r.l., Milan, Italy). Cylindrical shape scaffolds with a diameter of 6 mm and height of 4 mm (volume = 113 mm^3^) were obtained through a sterile biopsy punch (B Life S.r.l., Treviso, Italy) and seeded with 110 µL of chondrocyte suspension at a density of 1 × 10^6^ cells/mL. Relative luminescence units (RLUs) were recorded with a LuMate^®®^ luminometer (Awareness Technology Inc., Palm City, FL, USA) and the resulting data were analyzed through LuMate Manager software.

### 2.9. Chondrocyte 2D Culture

Human chondrocytes (commercial source, Cod. 402-05a, Cell Applications, Inc., San Diego, CA, USA) were seeded in 6-well plates at a density of 1.7 × 10^3^ cells/cm^2^ with 2 mL of growth medium and incubated at 37 °C in a 5% CO_2_ humidified incubator. To prepare chondrogenic medium, DMEM/F12 medium was supplemented with FCS 10%, penicillin-streptomycin 0.1 mg/mL, ascorbate-2-phosphate 50 μg/mL, TGF-β3 10 ng/mL, dexamethasone 20 μg/mL, 5 mL ITS (Insulin, Transferrin, Selenium), and Na-pyruvate 0.1 mg/mL (Sigma-Aldrich Corp., St. Louis, MO, USA). Cell morphology and distribution were monitored daily through an optical microscope for an overall period of 10 days.

### 2.10. Chondrocyte 3D Culture 

Cylindrical scaffolds were obtained from rectangular collagen membranes by pressing and rotating a circular 6 mm diameter biopsy punch (Kai Medical Europe, GmbH, Germany) on the surface under sterile conditions. Each scaffold had a diameter of 6 mm and was 5 mm thick, thus its volume was about 141 mm^3^. 1.5 × 105 human chondrocytes in 140 μL of growth medium were seeded on each cylindrical scaffold with a tuberculin syringe (Sigma-Aldrich Corp., St. Louis, MO, USA). Every single scaffold was placed in a well of a 24-well plate and incubated for 48 h at 37 °C in a 5% CO_2_ humidified incubator, to enhance cell adhesion; then, samples were inserted in the 3D KUBE^TM^ perfusion bioreactor system (3D Cell culture plasticware Kiyatec Inc., Pendleton, SC, USA) and 10 mL of chondrogenic medium were injected into each tube of the apparatus through 22 μm filters to avoid contamination, and the bioreactor was finally placed in the standard incubator at 37 °C and connected to the external pump that was programmed to push the medium at a speed of 60 μL/hour. The medium was completely replaced weekly.

### 2.11. RNA Isolation and Gene Expression Profiling

Type II collagen (*COL2A1*), SRY-Related HMG-BOX Gene 9 (*SOX9*), and Aggrecan (*ACAN*) were investigated as chondrogenic markers. Total RNA was extracted from chondrocytes at each time point using TRIAzol Reagent (Ambion by Life Technologies, Austin, TX, USA) and the DirectZol_RNA Mini Prep Kit (Zymo Research, Orange, CA, USA). For each sample, 1 μg of total RNA was reverse transcribed using the cDNA synthesis kit (Revert Aid RT Reverse Transcription Kit, K1691, Thermo Fisher Scientific, Waltham, MA, USA). Relative gene expression analysis was performed in a real-time PCR detection system (CFX96 TouchTM Real-Time PCR Detection System, Bio-Rad, Hercules, CA, USA), using the SensiFASTTM SYBR NO-ROX kit (Bioline, London, UK, Cat. No. BIO-98020) with the validated primers (Bio-Rad) and following MIQE guidelines [36]. Amplification was performed in a 10 μL final volume, including 2 ng of complementary DNA (cDNA) as a template. Triplicate experiments were performed for each explored condition, and data were normalized to Ribosomal Protein Lateral Stalk Subunit P0 (*RPLP*_0_) expression [37]. Fold change in gene expression was determined by the 2^−^^ΔΔCt^ method and presented as relative levels vs. untreated cells at time-zero.

### 2.12. Statistical Analysis

For comparison between two independent groups, a statistical analysis using the two-tailed independent Student’s *t*-test was performed. *p*-values < 0.05 were accepted as significant.

## 3. Results

The composite material combining equine type I collagen and hyaluronic acid was termed as HyCo in the following. 

**Figure 1:**
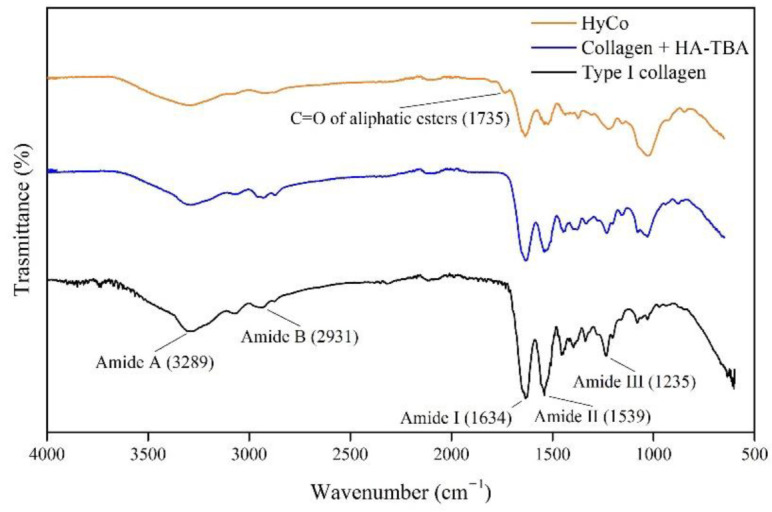
FT-IR spectra of commercial collagen scaffold and HyCo. All spectra were acquired at room temperature, using wavenumbers ranging from 650 to 4000 cm^−1^, with a resolution of 1 cm^−1^. The split signals of different samples are reported below. The representative peaks of major functional groups were assigned.

The lyophilized sponge of type I native heterologous collagen, extracted from the equine flexor tendon, was obtained from a commercial material (BIOPAD^®®^) largely recommended for the healing of connective tissues, including cartilage defects; it was hydrated with a water solution of hyaluronic acid and subsequently chemically crosslinked. Chemical and physical characterization was carried out in order to optimize synthesis protocol, adopting the commercial collagen scaffold as reference. Chemical bonds’ nature and differences in terms of functional groups between pure collagen and the novel crosslinked scaffold were investigated by FT-IR spectroscopy and the resulting spectra of all samples are shown in Figure 1. Specifically, the spectrum of commercial collagen scaffold showed traditional absorption bands of proteins, which are characterized by a predominant peak at a wavenumber about 1634 cm^−1^ for amide I (C=O stretching), followed by the presence of other two peaks at wavenumbers of 1539 and 1235 cm^−1^, associated with amide II (N-H deformation) and amide III (tertiary C-N stretching vibration) [38] respectively. Moreover, two other amide bands were evident at frequencies of 3289 cm^−1^ (amide A; N-H stretching) and 2931 cm^−1^ (amide B; C=O stretching).

In the case of the HyCo, a new peak in correspondence of the wavenumber of 1735 cm^−1^ appeared. Furthermore, all bands associated with amides were decreased in intensity after crosslink reaction (see also Figure S2).

### 3.1. Thermal Analysis

**Figure 2:**
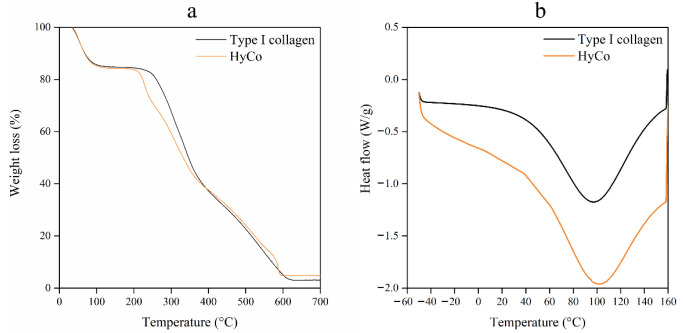
TGA and DSC analyses of commercial collagen scaffold and HyCo. TGA thermograms within the warming range from 0 to 700 °C (**a**); DSC profiles were recorded using a calorimeter with samples shielded in aluminum containers in the temperature range of −50 °C and 160 °C in nitrogen atmosphere. The DSC scan shows an endothermic peak at 100 °C attributed to water loss/evaporation (**b**).

Both thermal gravimetric (TGA) and differential scanning calorimeter (DSA) analyses were also performed. Figure 2a,b shows findings of them for collagen and HyCo scaffold. From TGA analysis, we obtained the curves associated with the mass loss (%) of samples during the warming process up to 700 °C. TGA curves of pure collagen and HyCo showed a similar profile with a stage of losing weight ranging from room temperature to 200 °C due to the evaporation of physisorbed water (Figure 2a). By analyzing curves’ trend, it should be noted that both scaffolds have hygroscopic properties; a further weight loss for both was shown between 250 and 550 °C related to the decomposition of collagen structure followed by finally slight loss between 550 and 700 °C resulting from the combustion of the residual organic components [39]. 

DSC data indicated different thermal behavior between commercial collagen scaffold and HyCo, as shown in Figure 2b. Indeed, they exhibited different endothermic peaks at 97.15 and 102.32 °C, respectively, and HyCo showed a unique and uniform DSC curve shape that was also used for quality control of cross-linked scaffolds. Furthermore, the denaturation temperature of HyCo was higher than that of the pure collagen. This suggests that the thermal stability of the collagen was enhanced by the presence of cross-linked hyaluronic acid; therefore, the reaction can be considered successful.

### 3.2. Morphological Analysis by FE-SEM

**Figure 3:**
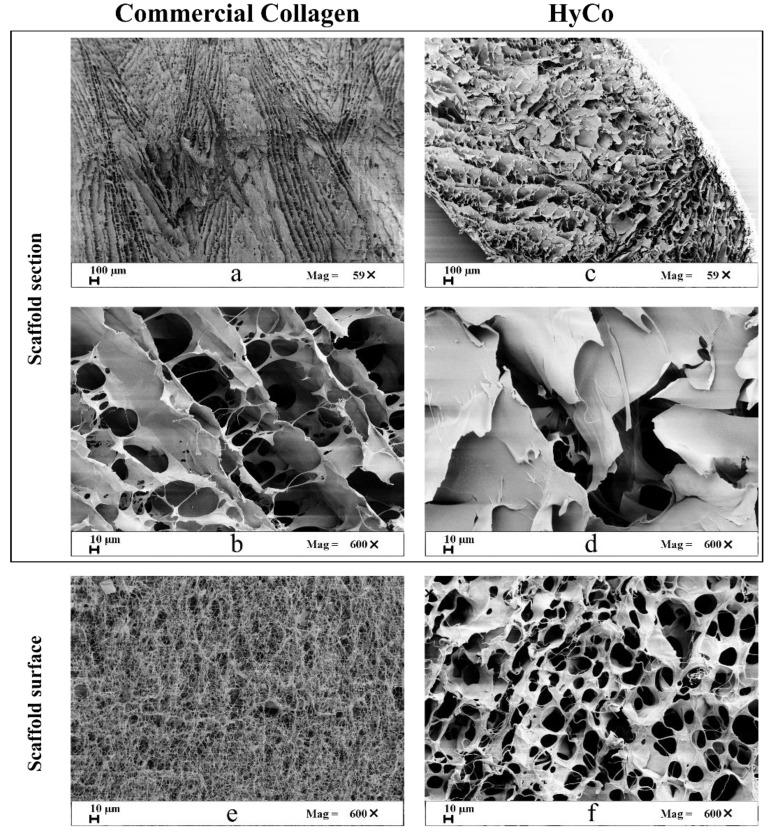
Comparison between SEM images of commercial equine type I collagen scaffold and HyCo. From the micrographs a different porosity and internal morphological organization of section and surface between commercial collagen (**a**,**b**,**e**) scaffold and HyCo one (**c**,**d**,**f**) is evident; however, both showed open and interconnected voids.

Samples were further characterized by field emission electron scanning microscopy to obtain information about their morphology and surface porosity. SEM images of the cross-sections of both scaffolds—obtained after cryo-sectioning—showed a highly porous structure (see Figure 3a,d) with a continuous network of open pores. In the case of commercial collagen scaffold, a dense random orienting fibers network, very slim in thickness was observed, especially on the surface (Figure 3e). In contrast, the cross-linking process with HA has changed the morphology and the porosity of HyCo, leading to a considerable thickening of the fibers as shown in Figure 3f. Finally, from digital analysis of SEM images, superficial pores of the sample after chemical reticulation had a round or oval shape with an average diameter about of 150 ± 70 μm.

### 3.3. Mechanical Characterization

**Figure 4:**
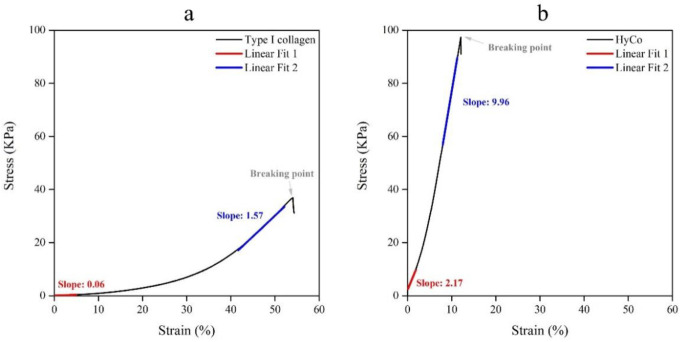
Stress–strain curves resulting from tensile tests of commercial collagen scaffold and HyCo and related Young’s Moduli. Type I collagen scaffold (**a**) and HyCo (**b**); HyCo scaffold displayed a significant statistical increase in both Young’s moduli with respect to the commercial collagen one. Slopes of straight lines were calculated on the linear portion of curves using least-squares fit on tensile test data by means OriginPro 2016 software (OriginLab, Northampton, MA, USA).

To evaluate differences in terms of mechanical behavior following cross-linking protocol, tensile tests in wet conditions (100% humidity) were performed. All scaffolds exhibited a similar stress–strain trend (see also Figure 4a,b); specifically, curves showed three distinct zones: a first linear region, termed ‘Toe’, in which stretching out of collagen fibers occurs in response to low mechanically loading; this zone ends under 10% of strain until all curled fibers straighten. Second linear region, in which the collagen fibers respond linearly to a higher load. Finally, irreversible failure. 

In the first linear region (Toe), collagen scaffold presented a Young’s modulus of 0.06 ± 0.04 kPa (see Table 1), while in the second linear one Young’s modulus reached a value of 1.57 ± 0.08 kPa. At the breaking point, the elongation and tensile strength were respectively 54.04 ± 8.20 and 36.91 ± 0.24. On the contrary, HyCo displayed a significant statistical increase in both Young’s moduli with respect to just collagen, achieving a value of 2.17 ± 1.35 (*p* ≤ 0.05) and 9.96 ± 0.53 kPa (*p* ≤ 0.001) respectively for the first linear region and the second one. In this last case, the percentage of elongation at break was notably decreased to 12.09 ± 1.16 whilst the resulting tensile strength raised to 97.36 ± 3.58.

### 3.4. Absorbency and Swelling Behavior

**Figure 5:**
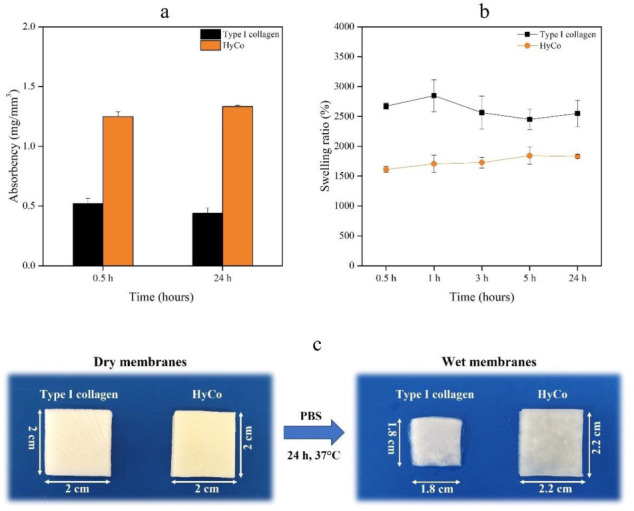
Absorbency and swelling ratio assays on commercial collagen scaffold and HyCo. Absorption capability of a saline solution (142 mM NaCl, 2.5 mM CaCl_2_) by collagen and HyCo scaffold at 30 min and 24 h expressed as mg/mm^3^ (**a**), and related swelling ratio (%) at different time points (**b**). Data are presented as mean ± standard error. Pictures are of dry samples and samples after 24 h of incubation with PBS at 37 °C (**c**).

HyCo and commercial collagen scaffold were tested to evaluate their ability in absorbing and retaining a saline solution at body temperature over time, according to a protocol described in the British Pharmacopoeia (1995) for textiles aimed at medical purposes [33]. The resulting absorptive capacities after 30 min and 24 h of incubation at 37 °C are shown in Figure 5a. From the comparison between the two kinds of scaffolds, it is evident that the absorption capability of the hybrid scaffold (HyCo) is higher compared to the collagen scaffold, as confirmed by absorbency values both after 30 min and 24 h. Indeed, commercial collagen was the least hydrated scaffold, absorbing 0.52 ± 0.04 mg/mm^3^ and 0.44 ± 0.04 mg/mm^3^ of saline solution after 30 min and 24 h respectively. On the contrary, HyCo showed the highest values, reaching 1.25 ± 0.04 mg/mm^3^ after 30 min and 1.33 ± 0.01 (*p* ≤ 0.0001) mg/mm^3^ after 24 h. 

The swelling ratios (%) in PBS (pH 7.4) related to both scaffolds were also determined, and the findings are shown in Figure 5b. It was observed that commercial collagen exhibited a rapid absorption capability, achieving the highest value of swelling ratio 2848 ± 266% after 1 h. However, this parameter tended to markedly decrease over time, reaching a percentage of 2550 ± 222 following 24 h. Differently, HyCo achieved the highest swelling ratio at 5 h with a percentage of 1842 ± 143 (*p* ≤ 0.05), showing a more rising and reproducible trend over 24 h, as confirmed by the low standard deviations. 

Another important aspect to consider is that, following the hydration phase, the collagen scaffold tended to lose its original shape, thus reducing its size over time, as confirmed by Figure 5c. Conversely, HyCo seemed to maintain its native structure, demonstrating a good shape memory during the whole incubation time.

### 3.5. Enzymatic Degradation and Cytotoxicity Assay

**Figure 6:**
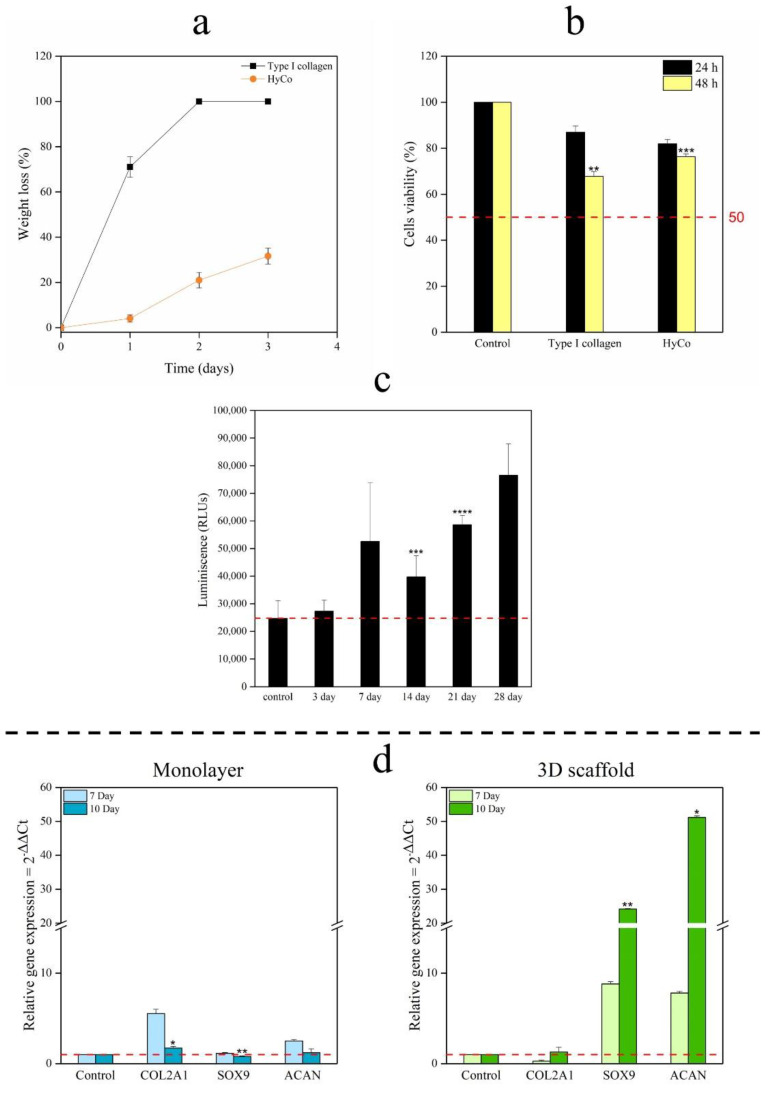
Degradation profiles of commercial collagen scaffold and HyCo, cytotoxicity study, and gene expression profiles. Comparison between enzymatic degradation of collagen and HyCo following treatment with collagenase from *Clostridium hystolyticum*, at 37 °C (**a**); Collagen and HyCo scaffolds cytotoxicity evaluated with human peripheral blood mononuclear cells (hPBMCs) by MTT assay: the histograms report the mean percentage of viable cells compared to controls of untreated cells (**b**). ATP activity of human chondrocytes cultured on HyCo scaffold for 28 days evaluated as mean of relative luminescence units compared to control. Experiments were analyzed by two-tailed Student’s *t*-test, * *p* ≤ 0.05, ** *p* < 0.01, *** *p* < 0.001 and **** *p* ≤ 0.0001; N = 3 (**c**); mRNA expression levels of chondrogenic markers (COL2A1, SOX9, and ACAN) of human chondrocytes cultured on 3D scaffold and 2D standard culture monitored by RT-qPCR; untreated cells at time zero were considered as control. * *p* < 0.05, ** *p* < 0.01 (two-way ANOVA). N = 3 biological replicates (**d**).

With the aim to evaluate kinetic profiles of samples, in vitro enzymatic degradation test was performed, using a type I collagenase derived from *Clostridium histolyticum*. In more detail, degradation behavior in the presence of collagenase at 37 °C (pH 7.4, 1.2 U/mL) is reported in Figure 6a. The results indicated that uncrosslinked commercial collagen was quickly degraded, losing 71 ± 4.5% of its mass in just 24 h; whilst the cross-linked one has lost 4.1 ± 1.6% of its weight after the same time, demonstrating a higher resistance to enzymatic activity. After 72 h of incubation with collagenase solution, the uncrosslinked scaffold was completely degraded. In contrast, HyCo showed a final degradation rate of 31.64 ± 3.5%. 

Furthermore, the cytotoxicity effects of scaffolds were explored on hPBMCs isolated from healthy donors to provide preliminary safety evidence directly on human primary cells [40]. To this aim, cells were incubated for 24 and 48 h along with scaffold samples weighing 10 mg (final concentration within each well was about 6.7 mg/mL), and their metabolic activity was analyzed by MTT assay (Figure 6b). Results demonstrated that, despite the high concentration tested, uncrosslinked commercial collagen has a very mild cytotoxic effect on hPBMCs, showing metabolic activity of 86.93 ± 2.8% and 67.81 ± 2.02% (*p* ≤ 0.01), respectively after 24 and 48 h. HyCo has shown a similar trend, achieving 81.92 ± 1.9% following 1 day and 76.37 ± 1.2% (*p* ≤ 0.001) after 48 h. 

Human chondrocytes were also cultured on HyCo scaffold for 28 days and their cell viability was monitored by an adenosine-5′-triphosphate (ATP) assay. From experimental data, a significant statistical increase can be observed in cell viability after 14 and 21 days of culture compared to control (cells just seeded), as documented in Figure 6c. Additionally, RT-qPCR analysis of the main genes associated with chondrogenic phenotype such as COL2A1, SOX9, and ACAN was performed with human chondrocytes cultured on the 3D scaffold for up 10 days under dynamic conditions using a perfusion bioreactor to ensure a more uniform nutrient distribution, as documented elsewhere [14]. 2D standard culture on flask was used as control. Our findings demonstrated that monolayer chondrocytes tend to lose their phenotype, as confirmed by the decrease in chondrogenic markers from Day 7 COL2A1 (5.52-fold), SOX9 (1.14-fold), and ACAN (2.50-fold) to Day 10 COL2A1 (1.73-fold; *p* ≤ 0.05), SOX9 (0.78-fold; *p* < 0.01), and ACAN (1.20-fold); in contrast, 3D scaffold was able to preserve the chondrocyte phenotype as documented by significant upregulation of all genes from Day 7 COL2A1 (0.26-fold), SOX9 (8.80-fold), and ACAN (7.80-fold) to Day 10 COL2A1 (1.29-fold), SOX9 (24.17-fold; *p* < 0.01) and ACAN (7.80-fold; *p* ≤ 0.05) (Figure 6d).

## 4. Discussion

Development of innovative biomaterials with suitable mechanical properties for in vivo application in cartilage defects is still a challenge [41]. Current management of cartilage lesions often requires invasive surgical procedures [42], which could be enhanced in terms of outcomes and prognosis by the development of advanced scaffolds [43]. In this context, type I collagen is one of the most employed natural proteins as a commercial scaffold available on the market, owing to its in vivo biodegradability and full safety as well as low antigenic potential [44], and for this reason, we have selected it for our experimentation. Moreover, despite the possibility of extracting collagen from different animal sources—including bovine and porcine sources—equine source is the most used as it is almost free from zoonosis risk [45]. Furthermore, from a clinical point of view, this material presents several limitations including high susceptibility to enzymatic degradation by collagenases, structural instability following hydration, and poor mechanical resistance; all these drawbacks decreased successful clinical outcomes significantly, thus discouraging its application. 

Departing from these weaknesses, we described here a novel process for synthesizing hybrid scaffolds (HyCo) by combining hyaluronic acid to equine type I collagen to obtain a composite material with improved physicochemical and biomechanical properties. It has to be underlined that the choice of the two biopolymers for HyCo synthesis was due to the consideration that cartilage is formed by collagen and proteoglycans that are macromolecules composed of a protein backbone (core) to which long chains of glycosaminoglycans (GAGs) are covalently linked; whereas, GAGs can be sulfated (such as chondroitin and keratan) or non-sulfated (as in the case of hyaluronic acid). As a consequence, both GAG and HA has the ability of retaining a high amount of water providing the greater mechanical strength of cartilage tissues. In pathological conditions, collagen fibrils are degraded and proteoglycan aggregates are cleaved from the hyaluronic acid backbone, leading to the deterioration of the physiological network with the loss of its functionality (see also Figure S3) [46]. Therefore, intending to mimic and restore the natural environment where chondrocytes reside, we decided to set up a novel hybrid scaffold using the combination of the two principal components of which cartilage ECM is composed. 

As already reported in the introduction, one of the major problems to tackle in synthesizing hybrid scaffolds based on hyaluronic acid and collagen is represented by the association of these two components. As is widely known, they have different behavior in terms of stability and in aqueous solution tend to generate polyion complexes, which interfere with proper scaffold structuring [30]. To overcome this, an incorporation process by heterogeneous phase was developed, starting from a collagen sponge and a hyaluronic acid water solution. In other words, the collagen sponge was first soaked with HA solution and then freeze-dried. The resulting co-lyophilized sponge then underwent a chemical cross-linking through activation of carboxylic functions to entrap HA within the collagen matrix (Figure S1), thus avoiding its early dissolution in contact with biological fluids [47]. It should be noted that the development of scaffolds by freeze-drying processes is very advantageous in the pharmaceutical field because water is the only medium employed as porogen before freezing [48]. Moreover, this process gives a highly porous structure to materials, boosting their hydrophilicity. This is a very important aspect to consider since all materials designed as scaffolds for use in cellular therapies must be porous to accommodate cells growth and proliferation. Furthermore, the porosity also impacts matrix permeability, which is required for exchanges of nutrients or waste products between the scaffold and external environment [49]. 

Among the described protocol, HA loading can be varied in the hydration step making the process extremely advantageous in terms of versatility. However, it should be noted that when using high concentrated HA solution (>15 mg/mL) the higher viscosity of the liquid hampers the proper solution absorption into collagen sponge; therefore, higher percentages of HA loading are not suggested. Chemical characterization by FT-IR analysis indicated that cross-linking leads mainly to an esterification reaction as confirmed by the presence of a new peak associated with carbonyl stretch of aliphatic esters within FT-IR spectrum related to HyCo (Figure 1). Therefore, we hypothesized that HA could form ester-type bonds between its carboxylic groups and amino and/or hydroxyl groups of type I collagen, following reticulation. Amide bonds within peptides give rise to different infrared signals associated with bonds vibrations; however, in our case, there was no meaningful evidence of change in the spectra with HA addition with respect to just collagen. The only difference observed is that all bands related to amides were decreased in intensity after reaction with hyaluronic acid. All these signals can be related to the triple-helical conformation of collagen fibrils as reported elsewhere [50]; therefore, we supposed that our process did not significantly affect collagen structure. FTIR analyses also confirmed that no other chemical reagents were embedded into the cross-linked scaffolds after synthesis protocol. Degradation thermal profiles, reported in Figure 2a, documented a similar trend between scaffolds, especially in the first phase, confirming hygroscopic properties. However, the second and last stretch related to the HyCo curve seemed to differ slightly with respect to control, probably in response to HA incorporation within the collagen matrix. DSC analysis confirmed that the thermal stability of collagen was improved after the cross-link reaction, by the increase in temperature associated with the endothermic peak (Figure 2b). From SEM images, the surface and internal porosity of scaffolds seemed affected by cross-linking due to the formation of smaller void areas. Micrographs always showed an open and interconnected void structure, consisting of pores with an average diameter not smaller than 50 µm (Figure 3a–f) that is considered the boundary size for ensuring proper distribution and cell viability [51]. Therefore, scaffold architecture could be suitable for cell colonization. 

The more relevant data indicate that cross-linking process strongly increased the mechanical strength of the system as documented by a 10-fold (*p* ≤ 0.001) increase in Young’s modulus and tensile strength at break, with respect to pure collagen (Table 1; Figure 4a,b). This result is extremely relevant if considering the potential scaffold application as a cartilage substrate. However, despite improved elastic modulus following cross-linking, it is still two orders of magnitude lower than one of articular cartilage [52]. An effective way to further improve the mechanical properties of the scaffold could be the rise of cross-linking degree varying the synthesis protocol by including a chemical cross-linker or prolonging the reaction time. However, higher cross-linking degree could also lead to a reduced porosity, thus interfering with cell harvesting and viability. On the other hand, when the scaffold is implanted and colonized by stem cells or chondrocytes, the novel extracellular matrix integrated within could enhance its mechanical behavior over time, partially offsetting the estimated gap.

Concerning absorption capability, it is well-known that collagen-based materials are easily wettable by aqueous solvents—including biological fluids—for the presence of many polar functional groups capable of binding water. Specifically following the hydration phase, these polymeric materials can swell and maintain a fraction of water within their structure over time. On the other hand, swelling properties of materials have been demonstrated to significantly affect cell behavior—including adhesion and proliferation. It should be noted that, after 24 h, a low change in weight of absorbed saline solution by both tested scaffolds was recorded, suggesting that their maximum hydration degree was reached in a short time. However, the hybrid scaffold has shown the highest absorbency value after 24 h (Figure 5a). Generally, the ability of water uptake by spongy scaffolds depends mainly on two factors: the hydrophilicity of material and the stability of its porous structure following water soaking. In more detail, the hydrophilicity of scaffolds is influenced by the following factors: the polarity of each macromolecular constituent and their crosslinks degree [48]. The presence of HA in scaffolds should promote intermolecular bonds by creating ion complexes between its carboxylic groups and the amino groups of collagen, thus diminishing the swelling ability. Furthermore, HA could also form ester-type bonds between its carboxylic groups and/or the hydroxyl groups of collagen, in the presence of cross-linkers, in this way providing covalent attachment of HA to collagen. From the analysis of swelling behavior it has been found that cross-linking causes a reduction in this parameter and suggests that a most stable network has been reached following this reaction (Figure 5b). Another important observation to consider is that, following the hydration phase, collagen tended to lose its original shape over time; this behavior might reduce its efficacy during in vivo applications. In contrast, HyCo seemed to maintain its native structure, demonstrating a good shape memory during the whole incubation time (Figure 5c). Therefore, we hypothesized that cross-linked scaffolds could better maintain their structural integrity in biological fluids following in vivo implantation. 

A further issue associated with the clinical failure of collagen-based scaffolds is their extreme susceptibility to enzymatic degradation by collagenases, which makes its catabolism faster. For this reason, we decided to also perform an in vitro enzymatic assay. From findings, it is evident that HyCo degraded more slowly with respect to collagen as such (Figure 6a). This peculiar behavior can be ascribed to cross-linking reaction with hyaluronic acid. Therefore, an increase in the structural stability of collagen by crosslinking reaction has proved a successful way to prevent catabolism by collagenase. Finally, to estimate if the crosslinking reaction may affect cytotoxicity of the material, in vitro cytotoxicity by using both human PBMCs and human chondrocytes was performed. Our data suggested that hybrid scaffolds still keep good biological characteristics after crosslinking and ensured good cell vitality. Indeed, a value of 82% of PBMC vitality after 24 h is extremely interesting taking into account the potential residues present in the scaffold structure after all the reaction steps. Furthermore, chondrocytes showed even more than 100% of viability after 72 h confirming the low toxicity of the described material. Finally, findings related to gene expression profiles of human chondrocytes suggested that our scaffold can preserve chondrogenic phenotype, as documented by a significant upregulation of *SOX9* and *ACAN* after 10 days of culture compared to the monolayer approach (Figure 6d) used as reference.

## 5. Conclusions and Perspectives 

In this study, a novel hybrid scaffold—based on collagen and hyaluronic acid—was successfully synthesized by a chemical crosslinking reaction performed in the heterogeneous phase. The novel scaffold showed strongly improved mechanical properties and higher resistance to enzymatic degradation, as well as lower cytotoxicity compared to commercial collagen. On the other hand, the described synthesis procedure is extremely versatile and can be largely applied to different collagen structures. Moreover, HyCo properties and its biomimetic structure seemed extremely interesting and may be further investigated within an in vitro and in vivo study to better ascertain its potential use as an adjuvant for treating cartilage defects. 

## Figures and Tables

**Table 1:** Young’s modulus, stress at break, and elongation at break of commercial collagen scaffold and HyCo.

	Type I Collagen	HyCo
Humidity (%)	100	100
Young’s modulus of TOE region (kPa)	0.06 ± 0.04	2.17 ± 1.35
Young’s modulus of linear region (kPa)	1.57 ± 0.08	9.96 ± 0.53
Elongation at break (%)	54.04 ± 8.20	12.09 ± 1.16
Tensile strength at break (kPa)	36.91 ± 0.24	97.36 ± 3.58

## Data Availability

Not applicable.

## Supplementary Materials

The following supporting information can be downloaded at: https://www.mdpi.com/article/10.3390/pharmaceutics14091752/s1, Figure S1: Schematic illustration of all process steps for synthesizing a composite scaffold. A type I collagen sponge is hydrated by HA-TBA water solution and follow undergone to freeze-dry for obtaining a co-lyophilized product. At this point, chemical cross-linking between collagen and HA is carried out through a reaction in heterogeneous phase. After chemical reticulation, the hybrid sponge is subjected to the final freeze-dry that leads to HyCo. Figure S2. FT-IR profiles for all the scaffold systems studied. All spectra were acquired at room temperature, using wavenumbers ranging from 650 to 4000 cm^−1^, with a resolution of 1 cm^−1^. The split signals of different samples are reported below. The representative peaks of major functional groups were assigned. Figure S3. Schematic illustration of cartilage extracellular matrix. Changes from healthy (A) to pathological conditions (B) are in the collagen fibrils that are degraded and in proteoglycan aggregates, which are cleaved from the hyaluronic acid backbone leading to the deterioration of the physiological network with the loss of its functionality (Figure adapted from Primorac et al., 2020, doi:10.3390/genes11080854, under Creative Commons Attribution (CC BY) license).
