# Supplementary material for: Synthesis and Characterization of a Novel Composite Scaffold Based on Hyaluronic Acid and Equine Type I Collagen
Source: Pharmaceutics. 2022 Aug 23;14(9):1752. doi: 10.3390/pharmaceutics14091752 (PMC9505875; doi:10.3390/pharmaceutics14091752)
Supplement: Supplementary file 1 [file pharmaceutics-14-01752-s001.zip › pharmaceutics-1799336-supplementary.pdf]

pharmaceutics

Article

Supplement materials: Synthesis and Characterization of a Novel Composite Scaffold Based on Hyaluronic Acid and Equine Type I Collagen

Erwin Pavel Lamparelli, Veronica Casagranda, Daniele Pressato, Nicola Maffulli, Giovanna Della Porta, and Davide Bellini

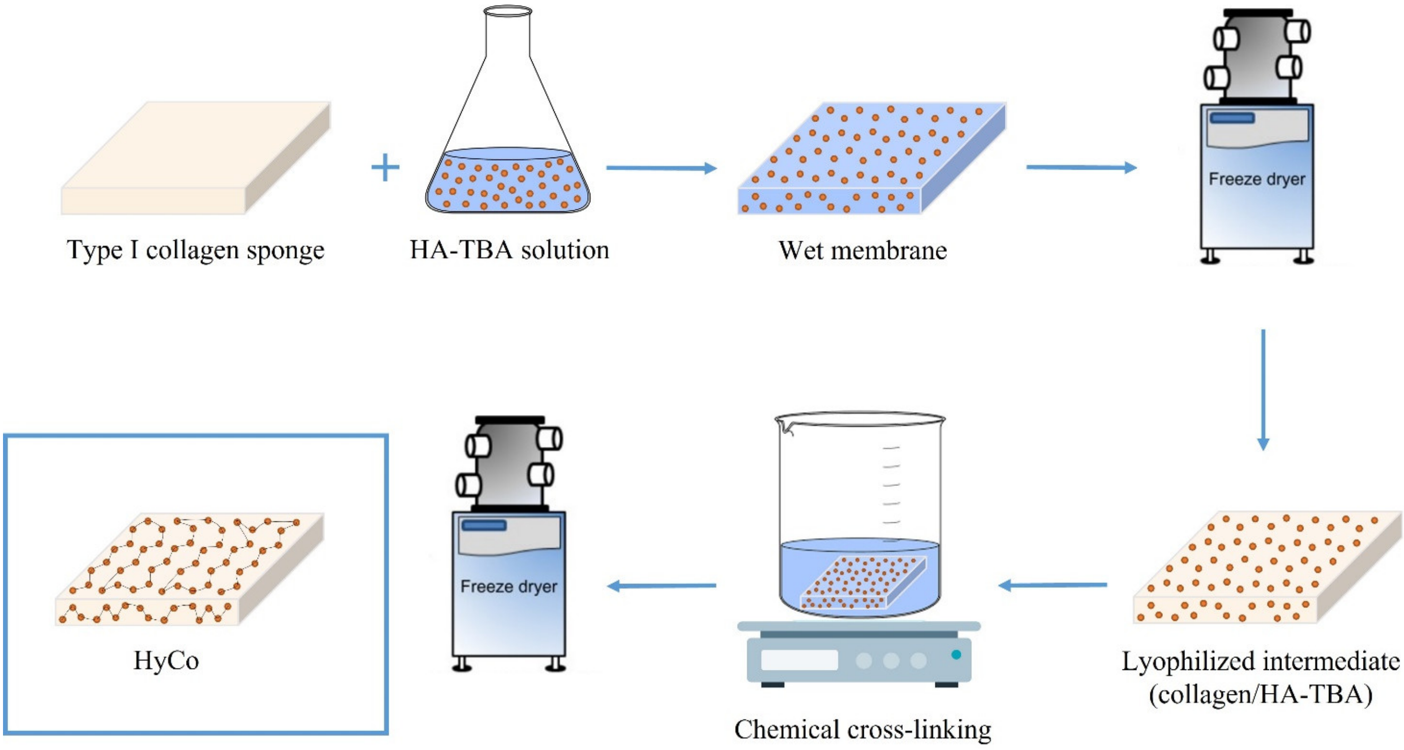

Figure S1. Schematic illustration of all process steps for synthesizing a composite scaffold.
A type I collagen sponge is hydrated by HA-TBA water solution and follow undergone to freeze-dyer for obtaining a co-lyophilized membrane. At this point, chemical cross-linking between collagen and HA is carried out through a reaction in heterogeneous phase. After chemical reticulation, the hybrid sponge is subjected to the final freeze-dryer that leads to HyCo.

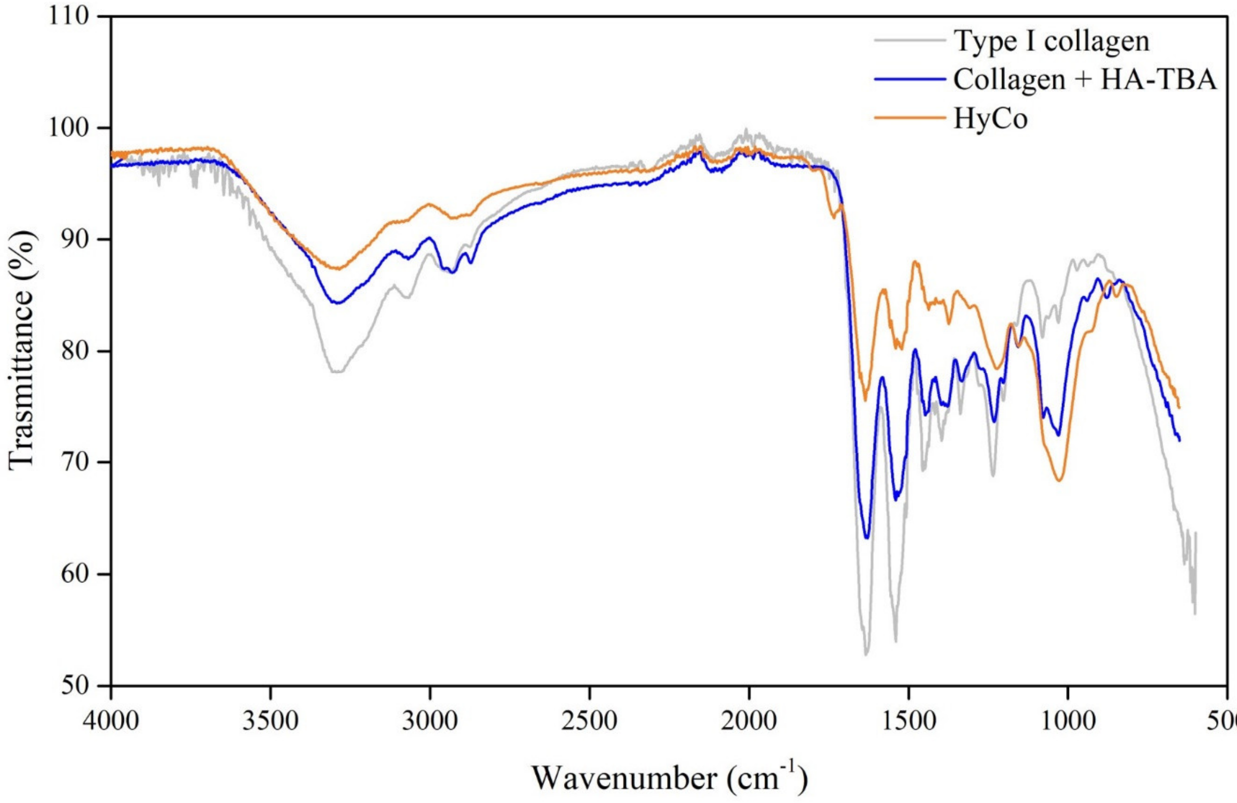

Figure S2. FT-IR profiles for all the scaffold systems studied.

All spectra were acquired at room temperature, using wavenumbers ranging from 650 to 4000 cm^{-1}, with a resolution of 1 cm^{-1}. The split signals of different samples are reported below. The representative peaks of major functional groups were assigned.

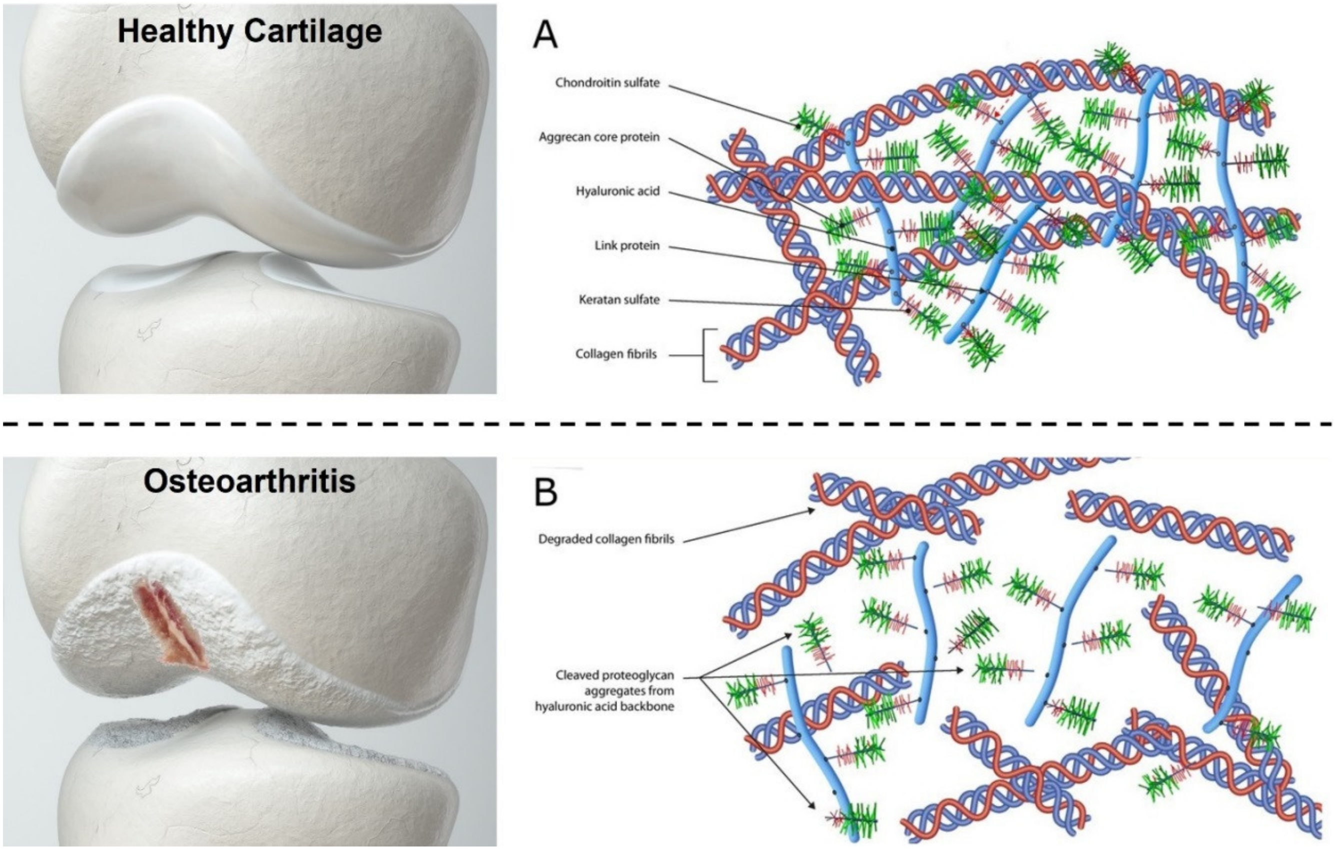

Figure S3. Schematic illustration of cartilage extracellular matrix.
Changes from healthy (A) to pathological conditions (B) are in the collagen fibrils that are degraded and in the proteoglycans aggregates, which are cleaved from the hyaluronic acid backbone leading to the deterioration of the physiological network with the loss of its functionality (Figure adapted from Primorac et al., 2020, doi:10.3390/genes11080854, under Creative Commons Attribution (CC BY) license).
